# Are preoperative CT variables associated with the success or failure of subsequent ventral hernia repair: nested case-control study

**DOI:** 10.1007/s00330-022-08701-x

**Published:** 2022-03-29

**Authors:** Shankar Kumar, Nikhil Rao, Sam Parker, Andrew Plumb, Alastair Windsor, Sue Mallett, Steve Halligan

**Affiliations:** 1 UCL Centre for Medical Imaging, University College London UCL, Charles Bell House, 43-45 Foley Street, London, W1W 7TS UK; 2 Radiology Department, University Hospitals Coventry and Warwickshire NHS Trust, Clifford Bridge Road, Coventry, CV2 2DX UK; 3 Addominal Wall Reconstruction Unit, University College London Hospital, 235 Euston Road, London, NW1 2BU UK

**Keywords:** Hernia, Hernia, ventral, Abdominal wall, Abdominal muscles, Tomography, spiral computed

## Abstract

**Objectives:**

Systematic review of CT measurements to predict the success or failure of subsequent ventral hernia repair has found limited data available in the indexed literature. To rectify this, we investigated multiple preoperative CT metrics to identify if any were associated with postoperative reherniation.

**Methods:**

Following ethical permission, we identified patients who had undergone ventral hernia repair and had preoperative CT scanning available. Two radiologists made multiple measurements of the hernia and abdominal musculature from these scans, including loss of domain. Patients were divided subsequently into two groups, defined by hernia recurrence at 1-year subsequent to surgery. Hypothesis testing investigated any differences between CT measurements from each group.

**Results:**

One hundred eighty-eight patients (95 male) were identified, 34 (18%) whose hernia had recurred by 1-year. Only three of 34 CT measurements were significantly different when patients whose hernia had recurred were compared to those who had not; these significant findings were assumed contingent on multiple testing. In particular, preoperative hernia volume (recurrence 155.3 cc [IQR 355.65] vs. no recurrence 78.2 [IQR 303.52], *p *= 0.26) nor loss of domain, whether calculated using the Tanaka (recurrence 0.02 [0.04] vs. no recurrence 0.009 [0.04], *p *= 0.33) or Sabbagh (recurrence 0.019 [0.05] vs. no recurrence 0.009 [0.04], *p *= 0.25) methods, differed between significantly between groups.

**Conclusions:**

Preoperative CT measurements of ventral hernia morphology, including loss of domain, appear unrelated to postoperative recurrence. It is likely that the importance of such measurements to predict recurrence is outweighed by other patient factors and surgical reconstruction technique.

**Key Points:**

• *Preoperative CT scanning is often performed for ventral hernia but systematic review revealed little data regarding whether CT variables predict postoperative reherniation*.

• *We found that the large majority of CT measurements, including loss of domain, did not differ significantly between patients whose hernia did and did not recur*.

• *It is likely that the importance of CT measurements to predict recurrence is outweighed by other patient factors and surgical reconstruction technique*.

## Introduction

Ventral hernia surgery is increasingly common, necessitated by rising obesity and abdominal surgery rates, both of which trigger subsequent hernias [1]. Repair of large hernias requires extensive abdominal wall reconstruction via mobilisation of tissue flaps that allow access to surgical planes for component separation, accompanied by prosthetic mesh implantation [2]. Reconstruction aims to cover the fascial defect, reapproximate the rectus muscles, and restore abdominal wall integrity. Extensive reconstruction is increasingly performed at specialised hernia centres, whose outcomes surpass “general” units. Nevertheless, even then, hernia recurrence following reconstruction approaches 40% [3, 4]. Accordingly, the ability to predict which patients may not benefit from surgery would have significant clinical utility.

A systematic review suggests that CT is underutilised prior to abdominal wall reconstruction [5]. Although CT can characterise preoperative morphology of abdominal wall musculature, when performed, imaging is often relegated to simple descriptions of the diameter and location of abdominal wall defect(s), and hernia content. A 2021 systematic review and meta-analysis identified predictors of postoperative recurrence, including patient variables (e.g. excessive BMI, female sex) and co-morbidities (e.g. smoking, diabetes, pulmonary disease) but found that neither hernia width nor area was associated significantly with recurrence [6]. However, the review found insufficient evidence to meta-analyse other CT variables potentially associated with recurrence. For example, CT can determine “loss of domain,” a surgical metric that describes the volumetric relationship between the hernia sac and residual abdominopelvic cavity. Two methods are used, Tanaka [7] and Sabbagh [8], but reported data were insufficient for meta-analysis [6]; Tanaka is the ratio when hernia sac volume is divided by residual abdominopelvic cavity volume whereas Sabbagh describes the proportion of total abdominopelvic cavity volume contained within the hernia. Accordingly, we investigated multiple preoperative CT metrics to identify if any were associated with postoperative reherniation.

## Methods

We developed a protocol in advance. Ethical permission was obtained to access patient data from our local abdominal wall reconstruction unit, and to contact patients by telephone where necessary, thereby obtaining verbal consent. The sample size was pragmatic: via departmental database review, we identified all patients undergoing abdominal wall reconstruction to treat VH from 1^st^ January 2008 to 31^st^ December 2017 inclusive, and the date of their surgery. We excluded lateral and parastomal hernias since they are aetiologically distinct and they recur more frequently than midline hernia. We then cross-referenced imaging data to identify those patients who underwent preoperative CT scanning within the 6 months preceding surgery. Imaging was retrieved; individual patients assigned a random study number, anonymised, and transferred to a personal computer. Scanning was acquired following intravenous contrast (unless contraindicated), using a range of multidetector row machines collimated to no more than 1mm.

**Fig. 1:**
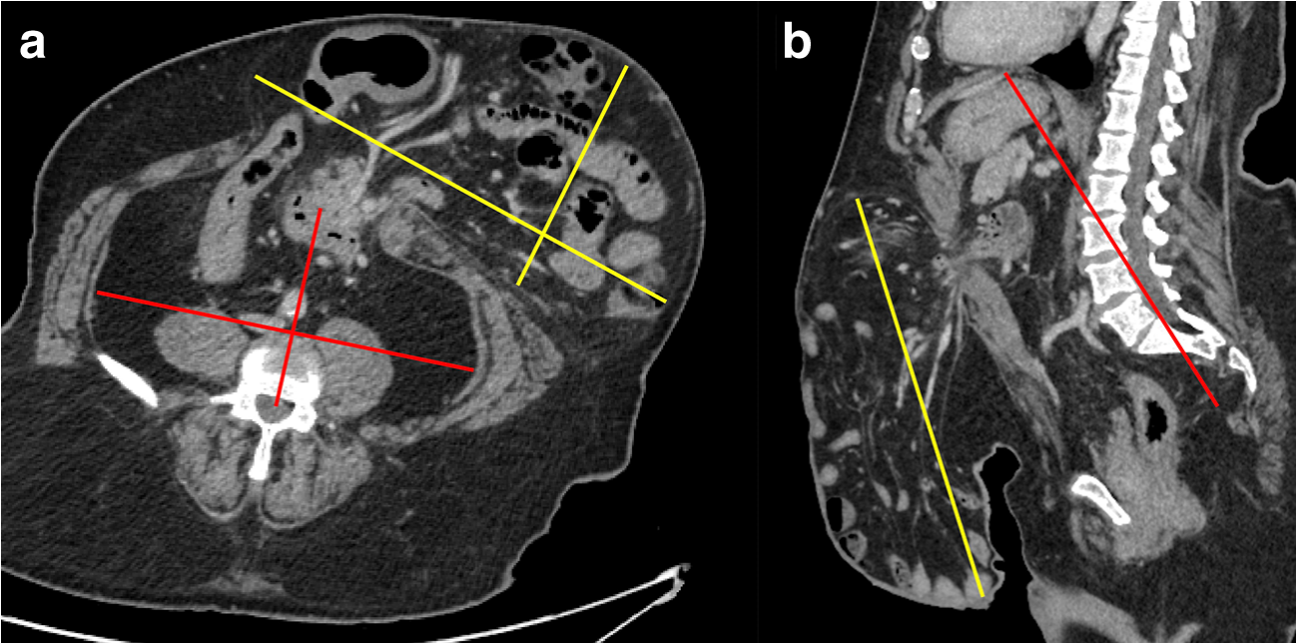
Axial (**a**) and sagittal (**b**) reconstructions of a patient with a complex ventral hernia showing the planes of linear measurements made to calculate volume ratios for loss of domain, for the hernia sac (yellow lines) and residual abdominopelvic cavity volume (red lines)

Ten patients (chosen randomly) were both measured by two radiology researchers (S.K., N.R.) to assess inter-reader agreement. This was deemed acceptable and the two researchers then examined half of the study cohort each, working independently. Radiologists were unaware of patient outcomes. Measurements were made using Horos (Version 3.3.6, Horos Project). The following variables were measured for each patient and extracted into a spreadsheet (Microsoft Excel for Mac version 16.48, Microsoft Corporation): hernia sac width, length, depth; residual abdominal cavity width, length, and depth were all measured as described by Tanaka, i.e. using the plane that demonstrated the maximum dimension for each individual measurement (Fig. 1) [7]. These data were used to calculate hernia and residual abdominal cavity volumes and thence Tanaka [7] and Sabbagh [8] volume ratios. We also recorded maximal axial rectus separation; craniocaudal extent of rectus separation; the distance this commenced below the xiphisternum and above the symphysis; hernia neck width. For both the rectus and strap muscle complex bilaterally, we recorded their maximal width and depth; their cross-sectional area; and mean Hounsfield measurement. We measured external abdominal circumference; cross-sectional area of subcutaneous and intra-abdominal fat (via segmentation tools). Measurements for axial orientations were made at the level of the greatest hernia defect width or abdominal girth, contingent on the measurement made. Software calipers were used to measure linear distance. The area was measured using a freehand region-of-interest (ROI) tool to outline the tissue of interest. Measurements were mm/mm^2^/Hounsfield units as appropriate. The strap muscle was defined as the muscular complex comprising the external and internal oblique, and the transversus abdominis collectively. In addition to the quantitative measurements described, observers described hernia content; whether they considered the rectus muscles attenuated/atrophic by subjective assessment (response yes/no); whether a mesh was present. We also extracted details of surgical technique, specifically whether a mesh was implanted and/or whether component separation was used.

Repair success or failure (defined as symptomatic recurrence) for individual patients at a 1-year postoperative time point was determined by a surgeon (S.P.) via both paper and electronic case note review. If outcome clarification was necessary, the surgeon telephoned the patient’s general practitioner and/or the patient themselves.

### Statistical analysis

We allocated patients to either “success” or “failure” groups, reported these data as median and inter-quartile range, and compared these using Mann-Whitney hypothesis tests, mindful of multiple comparisons. There were no missing data. A correlation matrix was used to assess linear associations between multiple variables. Significance was set at 5%. We used Bland-Altman analysis to assess inter-observer agreement [9]. Analysis was performed using Prism (version 9.1.2, Graphpad). We reported the research according to STROBE guidelines [10].

## Results

We reviewed data from 428 patients: 115 had no available CT scanning. Ninety-three were excluded due to inappropriate imaging (i.e. postoperative CT; incomplete abdominopelvic coverage; imaging outside preoperative temporal window). Outcome at 1-year could not be determined in 32. Ultimately, 188 patients had complete data for CT and outcome, 34 (18%, mean age 58, range 22 to 86; 73 male) whose hernia recurred within 1 year and 154 (82%, mean age 56, range 27 to 90; 22 male) who did not.

Of these, 14 presented to our centre with a primary ventral hernia (2 of whom recurred following our surgery), 117 an incisional hernia following surgery (13 of whom recurred following our surgery), and 52 a recurrent hernia following previous repair (14 of whom recurred following our surgery); these data were missing for 5 patients. Surgery was performed by a variety of surgeons working in a tertiary referral abdominal wall reconstruction unit. Of the 154 patients without recurrence, 139 (90%) had a mesh implanted and 12 (8%) did not (data missing for 3 patients) versus 23 (68%) and 5 (15%) respectively for the 34 who recurred (data missing for 6 patients). Fascial closure was achieved in 128 (83%) versus 20 (13%) patients who did not recur (data missing for 6 patients) versus 20 (60%) and 8 (24%) of those who recurred (data missing for 6 patients).

**Fig. 2:**
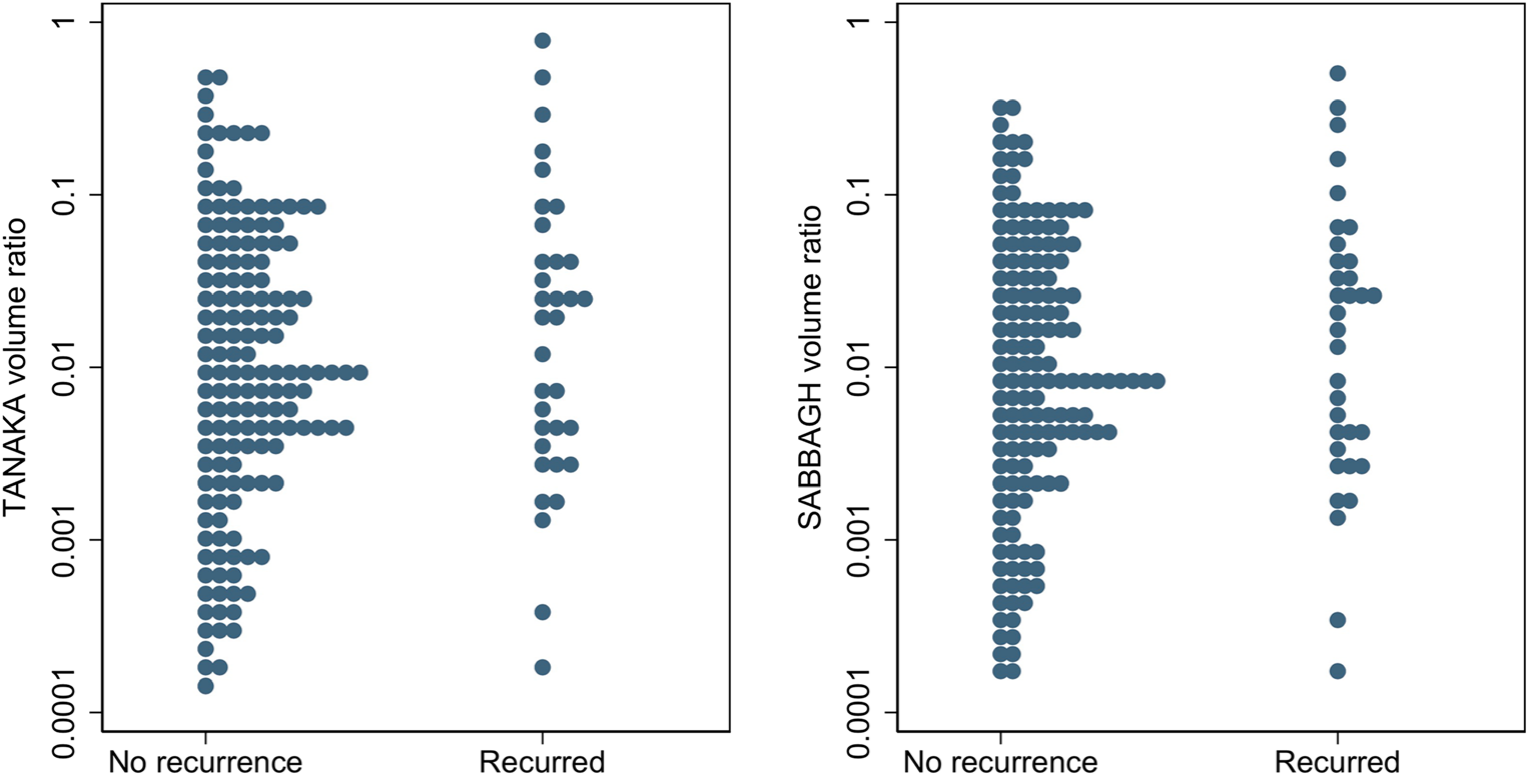
Dot plot showing the volume ratio (y-axis) for both the Tanaka method (left-sided plot) and Sabbagh method (right-sided plot) in patients whose ventral hernia recurred compared to those who did not

Table 1 details a comparison of quantitative measurements between the two groups for the hernia and residual abdominopelvic cavity, including loss of domain. Table 2 details a comparison of quantitative measurements between the two groups for the abdominal wall and its musculature. We made 34 individual group comparisons, of which only three were significantly different (left rectus Hounsfield measurement; right and left strap muscle area). Since the chance of at least one false-positive finding at 34 comparisons is 82.5% at the 5% level, these results may be spurious. However, it is interesting that both strap muscles appeared significant. The most noteworthy finding was that neither the Tanaka nor Sabbagh volume ratios were significantly different between patients whose hernia did and did not recur (Table 1, Fig. 2). As expected, these data were highly correlated with the linear measures from which they were derived (i.e. length, width, depth of both the hernia, and residual abdominopelvic cavity), and, accordingly, none of these differed significantly between groups (data not shown).

**Table 1:** Table comparing quantitative CT measurements of the hernia and abdominopelvic cavity of patients whose ventral hernia recurred (*n *= 34) compared with those who did not (*n *= 154)

				Bland-Altman agreement statistics			
Quantitative measurements	Recurrence: median (IQR)	No recurrence: median (IQR)	Probability* (p)	Mean measurement	Mean difference	Lower LOA	Upper LOA
Hernia sac width (mm)	79 (81.75)	72 (65.25)	0.30	80.6	0.9	− 5.9	7.7
Hernia sac depth (mm)	34.5 (26.25)	32 (23.75)	0.30	31.9	− 0.5	− 3.8	2.8
Hernia sac length (mm)	84.5 (94.75)	70 (93)	0.18	98.7	0.7	− 4.1	5.5
Hernia sac volume (mm^3^)	155,300 (355,650)	78,200 (303,520)	0.26	1,876,700	7300	− 31,400	46,100
Residual abdominal cavity width (mm)	280 (44.5)	273 (41)	0.33	261.5	2.7	− 4.5	9.9
Residual abdominal cavity depth (mm)	182 (35.75)	170 (42)	0.21	180.8	3.4	− 3.6	10.4
Residual abdominal cavity length (mm)	360.5 (40.75)	357 (42.5)	0.57	344.4	3.2	− 2.0	8.4
Abdominal cavity volume (mm^3^)	8,709,000 (4,223,150)	8,453,000 (3,971,290)	0.21	8,590,960	309,300	− 114,400	732,900
Loss of domain: Tanaka method	0.02 (0.04)	0.009 (0.04)	0.33	0.03	− 0.001	− 0.004	0.003
Loss of domain: Sabbagh method	0.019 (0.05)	0.009 (0.04)	0.25	0.03	− 0.001	− 0.004	0.002
Maximal rectus separation (mm)	66.5 (57.25)	65.5 (41.75)	0.17	79.9	1.9	− 5.7	9.5
Craniocaudal extent of rectus separation (mm)	81 (92)	62 (105.25)	0.08	91.5	0	− 7.7	7.7
Hernia distance below xiphisternum (mm)	131 (76.5)	140.5 (79.5)	0.33	119.3	2.7	− 2.3	7.7
Hernia distance above symphysis (mm)	147 (89)	147 (85)	0.80	143.8	2.6	− 2.2	7.4
Hernia neck width (mm)	66 (47)	59.5 (57.75)	0.12	78.2	1.6	− 6.4	9.6

*Mann-Whitney *U* test

*IQR* inter-quartile range

*LOA* limits of agreement

*HU* Hounsfield unit

**Table 2:** Table comparing quantitative CT measurements of the abdominal wall and musculature of patients whose ventral hernia recurred (*n *= 34) compared with those who did not (*n *= 154)

				Bland-Altman agreement statistics			
Quantitative measurements	Recurrence: median (IQR)	No recurrence: median (IQR)	Probability* (p)	Mean measurement	Mean difference	Lower LOA	Upper LOA
Right rectus maximal width (mm)	73 (31.75)	61 (24)	0.22	57.9	0.3	− 5.8	6.4
Right rectus maximal depth (mm)	12 (5.75)	11 (5)	0.51	13.0	0.2	− 1.1	1.5
Right rectus area (mm)	651.5 (446.25)	567 (331)	0.12	553.8	−2.5	− 29.5	24.5
Right rectus Hounsfield measurement (HU)	5 (40.5)	7.5 (44)	0.15	36	−1.6	− 7.5	4.3
Left rectus maximal width (mm)	60 (28.75)	61 (23)	0.54	62.7	0.3	− 3.5	4.1
Left rectus maximal depth (mm)	12 (4.75)	12 (4)	0.77	12.3	0.4	− 1.9	2.7
Left rectus area (mm^2^)	611.5 (394)	585 (317)	0.22	599	− 8.4	− 36.4	19.6
Left rectus Hounsfield measurement (HU)	−2 (24)	11 (35)	0.01	20.4	− 0.3	− 5.0	4.4
Right strap muscle maximal width (mm)	143 (55)	127.5 (81.5)	0.44	146	− 1.8	− 6.9	3.3
Right strap muscle maximal depth (mm)	24.5 (10.5)	22 (10)	0.25	20.1	0	− 2.7	2.7
Right strap muscle area (mm)	2763 (1067.5)	2055 (1420.25)	0.04	2353.9	− 108.1	− 670.4	454.2
Right strap muscle Hounsfield measurement (HU)	22 (21.25)	25 (28)	0.78	21.9	0.1	− 4.1	4.3
Left strap muscle maximal width (mm)	147 (73.75)	128 (82.75)	0.34	141	− 2.3	− 10.5	5.9
Left strap muscle maximal depth (mm)	24 (11)	23 (11)	0.35	21.2	0.8	− 1.8	3.4
Left strap muscle area (mm^2^)	2793 (1626.25)	2085 (1557.25)	0.02	2304.6	− 3.4	− 38	31.2
Left strap muscle Hounsfield measurement (HU)	20 (18.75)	25.5 (25)	0.16	23.9	− 0.5	− 5.6	4.7
External abdominal circumference (mm)	1108 (190.75)	1067 (208.25)	0.20	1799	− 2.8	− 69.4	63.8
Subcutaneous abdominal wall fat area (mm^2^)	26,573 (10,961.75)	23,533 (23,327.25)	0.53	21223	− 4.3	− 86.1	77.5
Intra-abdominal fat area (mm^2^)	18,180 (13,622)	15,420 (13,303.75)	0.20	13114	− 41.1	− 113.8	31.6

*Mann-Whitney *U* test

*IQR* inter-quartile range

*LOA* limits of agreement

*HU* Hounsfield unit

Concerning qualitative measurements, hernia contents were as follows: fat alone in 12 (35%) recurrence vs. 63 (41%) non-recurrence; small bowel in 12 (35%) recurrence vs. 39 (25%) non-recurrence; colon in 3 (9%) recurrence vs. 15 (10%) non-recurrence; both small bowel and colon 6 (18%) recurrence vs. 34 (22%) non-recurrence. One additional patient’s hernia contained liver and stomach along with bowel (who recurred) vs. three who did not recur (whose hernia contained liver, stomach, and bladder respectively). Both rectus muscles were considered morphologically normal in 14 (41%) patients who recurred vs. 67 (44%) who did not, whereas both rectus muscles were considered atrophic/attenuated in 17 (50%) patients who recurred vs. 75 (49%) who did not. A mesh was visualised with certainty in 2 patients who recurred and 13 who did not. Observers were unable to make a confident decision re-mesh presence/absence in 7 patients who recurred and 18 who did not.

Table 1 also details inter-reader agreement for the subset of patients measured by both observers. Generally, the mean difference and limits of agreement appear reasonable, suggesting that the analyses are not confounded to any great degree by inter-reader measurement error agreement was perfect for subjective impression of muscular atrophy/attenuation.

## Discussion

We examined preoperative CT studies from patients undergoing abdominal wall reconstruction to treat ventral hernia, in order to identify factors potentially associated with recurrence. Surgeons believe that increased loss of domain frustrates return of hernia contents to the abdomen and increases intra-abdominal pressure and fascial tension on closure, factors thought to precipitate recurrence. CT scanning estimates loss of domain by describing the relationship between hernia volume and residual abdominopelvic volume via “Tanaka” [7], or total intraperitoneal volume via “Sabbagh” [8]. We investigated ventral hernias of all sizes, hoping to identify a threshold ratio that might predict recurrence but we were surprised to find that preoperative measurements appeared unassociated with recurrence; specifically, larger hernias did not appear to recur more frequently.

 Authors have investigated the relationship between preoperative CT scanning and intra-operative outcomes, notably tension-free fascial closure. Franklin and colleagues [11] used logistic regression to identify CT factors associated with bridged repair, but with only 6 such patients from 54, their analysis was underpowered [12]. Fafaj and colleagues [13] investigated the Tanaka ratio in patients with a hernia width of at least 18cm, concluding that a ratio of more than 0.25 could not predict incomplete fascial closure reliably, i.e. difficult closure was not assured in large hernias. Schlosser and colleagues [14] investigated perioperative outcomes, notably respiratory failure, finding that a Tanaka ratio greater than 0.5 was associated with respiratory failure.

Other authors have investigated longer-term outcomes. Winters and colleagues [15] investigated both postoperative and longer-term complications, including postoperative recurrence. They measured several CT variables similar to our own, including loss of domain (method undescribed) in 65 subjects, 18 (28%) of whom recurred. While they found that visceral/subcutaneous fat volume and hernia sac volume predicted reherniation, by investigating 16 univariate predictors with just 18 recurrences, the analysis was likely to generate false-positive associations [12]. Our own study found no association between these variables and reherniation. Blair and co-workers [16] analysed abdominal wall thickness at umbilical level and defect size, finding no association with recurrence but with just 4 recurrent hernias, the study was also underpowered for regression methods.

Barnes and co-workers [17] found that preoperative sarcopenia, defined as < 19.6 HU measured from the psoas muscle, was associated significantly with postoperative recurrence (as a secondary outcome), which affected 11 of 58 patients. Again, since 14 variables were investigated using regression methods, the study appears underpowered. In contrast, Siegal and co-workers [18] found no such association in 135 patients, 39 of whom recurred, nor did Rinaldi and co-workers [19] in a series of 82, 17 of whom recurred. While our primary interest was not sarcopenia, we did find a significant relationship between muscle mass/attenuation, and subsequent recurrence (i.e. left rectus Hounsfield measurement; right and left strap muscle area). However, recurrence was associated with greater muscular cross-sectional area, leading us to conclude that the finding was due to multiple testing rather than a real association (and certainly not due to sarcopenia, which would reduce cross-sectional area). It is conceivable that it is a manifestation of the lateral muscular retraction and “bunching” that occurs in long-standing, large hernias, because it is known that without insertion into the linea alba, the strap muscle complex becomes less elastic, shorter, and thicker bilaterally [20]. It is also pertinent to note that had we formally applied Bonferroni’s correction (changing the significance threshold to 0.001), then no CT factors would have been significant.

In an attempt to synthesise outcomes from multiple, small, single-centre studies, a recent prognostic systematic review meta-analysed potential predictors of postoperative recurrence, including hernia width and area, but neither of these were associated significantly with reherniation [6]. We were interested in developing a multivariable model to predict hernia recurrence and performed the current study in order to identify CT factors that might usefully be incorporated in the development of a future model that also incorporated relevant clinical factors. We focused on reherniation at 1 year and investigated a wider range of potential CT measurements than examined previously. We are aware that many such measurements will be closely correlated with one another (hence we used a correlation matrix). Ultimately, our data suggest that CT measurements alone are unlikely to contribute usefully to any future multivariable model. While obtaining CT measurements, it was immediately apparent that the examples provided by Tanaka [7] and Sabbagh [8] in their original descriptions do not reflect the morphology of many ventral hernias encountered in daily practice. This may underpin why these measurements do not appear predictive. Most obviously, supine scanning encourages gravity to reduce hernia volume, meaning that CT estimates of volume may be less than when erect, and the estimated volume may not truly reflect disability. Similarly, while we noticed that many patients had widely separated rectus muscles, this was not necessarily associated with hernia sac protrusion. Prone scanning is unlikely to help (even when this can be achieved), since the abdominal wall is supported by the scanner table.

Our study does have limitations. A priori we believed its retrospective nature might introduce spectrum bias towards larger hernias. We did not believe this was problematic because we assumed larger hernias to be at greater risk of recurrence but, in reality, many of the patients identified had small hernias. Recurrence was defined by case note review and by contacting patients directly. While this approach will inevitably miss subclinical recurrence, our surgeons consider only symptomatic recurrence to be clinically important. We did not use uni- or multivariable regression techniques to investigate associations between CT findings and other potential predictors of recurrence because, while these methods have been used extensively in the indexed literature, studies are usually underpowered. With 34 recurrences, we had sufficient power to investigate just three predictors via regression and so used simple hypothesis testing instead, framing the research as a “predictor finding” exercise, confined to CT variables alone. Retrospective data such as ours are difficult to interpret because surgeons may have taken measures to combat recurrence in hernias that appear large by CT criteria, most obviously by using advanced component separation and mesh implantation techniques [2]. The precise effect of CT measurements on surgical decision-making can only be determined prospectively but our current findings suggest that, in isolation, they are unassociated with recurrence.

In summary, a comparison of multiple parameters obtained from preoperative CT scanning found few that were associated with postoperative recurrence. In particular, measurements of hernia morphology, including loss of domain, did not appear related to postoperative recurrence. It is likely that hernia volume and similar measurements are not useful when used in isolation to predict hernia recurrence, and their importance is outweighed by other patient factors and reconstruction technique.

## Abbreviations

CC: Cubic centimetre
CT: Computed tomography
HU: Hounsfield unit
IQR: Inter-quartile range
LOD: Loss of domain
STROBE: Strengthening the Reporting of Observational Studies in Epidemiology
VH: Ventral hernia

## Declarations

### Guarantor

The scientific guarantor of this publication is Steve Halligan.

### Statistics and biometry

One of the authors has significant statistical expertise.

### Methodology

• retrospective

• case-control study

• performed at one institution
